# Advantages of Tyrosine Kinase Anti-Angiogenic Cediranib over Bevacizumab: Cell Cycle Abrogation and Synergy with Chemotherapy

**DOI:** 10.3390/ph14070682

**Published:** 2021-07-16

**Authors:** Jianling Bi, Garima Dixit, Yuping Zhang, Eric J. Devor, Haley A. Losh, Andreea M. Newtson, Kristen L. Coleman, Donna A. Santillan, Thorsten Maretzky, Kristina W. Thiel, Kimberly K. Leslie

**Affiliations:** 1 Department of Obstetrics and Gynecology, University of Iowa, Iowa City, IA 52242, USA; Jianling-bi@uiowa.edu (J.B.); yuping-zhang@uiowa.edu (Y.Z.); eric-devor@uiowa.edu (E.J.D.); haley-losh@uiowa.edu (H.A.L.); andreea-newtson@uiowa.edu (A.M.N.); donna-santillan@uiowa.edu (D.A.S.); kimberly-leslie@uiowa.edu (K.K.L.); 2 Department of Internal Medicine, University of Iowa, Iowa City, IA 52242, USA; garima-dixit@uiowa.edu (G.D.); kristen-coleman@uiowa.edu (K.L.C.); thorsten-maretzky@uiowa.edu (T.M.); 3 Holden Comprehensive Cancer Center, University of Iowa, Iowa City, IA 52242, USA

**Keywords:** endometrial cancer, bevacizumab, cediranib, patient-derived organoid models, 3D culture, personalized medicine, p53, vascular endothelial growth factor (VEGF)

## Abstract

Angiogenesis plays a crucial role in tumor development and metastasis. Both bevacizumab and cediranib have demonstrated activity as single anti-angiogenic agents in endometrial cancer, though subsequent studies of bevacizumab combined with chemotherapy failed to improve outcomes compared to chemotherapy alone. Our objective was to compare the efficacy of cediranib and bevacizumab in endometrial cancer models. The cellular effects of bevacizumab and cediranib were examined in endometrial cancer cell lines using extracellular signal-related kinase (ERK) phosphorylation, ligand shedding, cell viability, and cell cycle progression as readouts. Cellular viability was also tested in eight patient-derived organoid models of endometrial cancer. Finally, we performed a phosphoproteomic array of 875 phosphoproteins to define the signaling changes related to bevacizumab versus cediranib. Cediranib but not bevacizumab blocked ligand-mediated ERK activation in endometrial cancer cells. In both cell lines and patient-derived organoids, neither bevacizumab nor cediranib alone had a notable effect on cell viability. Cediranib but not bevacizumab promoted marked cell death when combined with chemotherapy. Cell cycle analysis demonstrated an accumulation in mitosis after treatment with cediranib + chemotherapy, consistent with the abrogation of the G2/M checkpoint and subsequent mitotic catastrophe. Molecular analysis of key controllers of the G2/M cell cycle checkpoint confirmed its abrogation. Phosphoproteomic analysis revealed that bevacizumab and cediranib had both similar and unique effects on cell signaling that underlie their shared versus individual actions as anti-angiogenic agents. An anti-angiogenic tyrosine kinase inhibitor such as cediranib has the potential to be superior to bevacizumab in combination with chemotherapy.

## 1. Introduction

Endometrial carcinoma is the most common gynecologic malignancy in the United States. In 2020, over 65,000 women were diagnosed with endometrial cancer and nearly 13,000 women died of this disease [1]. Although the five-year survival rate of patients with early-stage endometrial cancer is relatively high, patients with advanced or recurrent endometrial cancer have a poor prognosis. Additionally, population studies have shown that endometrial adenocarcinoma is one of the only cancers in which prevalence and mortality are increasing [2]. Recent progress has been made in defining the molecular biology of endometrial carcinoma, which has led to the use of targeted agents to treat this disease. The addition of targeted therapies including anti-angiogenics in conjunction with chemotherapy may be more effective than chemotherapy alone, as we have recently reported [3].

The Gynecologic Oncology Group (GOG, now NRG Oncology) has evaluated a series of anti-angiogenic agents in recurrent endometrial carcinoma. Bevacizumab (Avastin^®^, Genentech) is a recombinant humanized antibody against vascular endothelial growth factor-A (VEGF-A), and it has been studied as a single agent in women with recurrent endometrial cancer in study protocol GOG-229E [4]. From this study, bevacizumab significantly improved PFS compared to additional treatment with chemotherapy [4]. GOG-229G, a Phase II trial of combination bevacizumab and temsirolimus (an mTOR inhibitor) was also deemed active in the treatment of recurrent endometrial carcinoma; however, the combination showed significant toxicity [5]. 

Cediranib is a small molecule multi-tyrosine kinase receptor inhibitor that targets VEGF receptors (VEGFR1/2/3), platelet-derived growth factor receptors (PDGFRα/β and c-Kit), and fibroblast growth factor receptor (FGFR1) [6,7]. GOG-229J was a study of cediranib as monotherapy in advanced endometrial carcinoma that, like bevacizumab, met the criteria for being considered an active agent in advanced endometrial cancer [8]. It was also found to be tolerable when administered as a single agent [8]. Other anti-angiogenic agents have also been investigated, but they have shown limited activity as single agents in unselected patients [9,10,11,12]. The GOG-86P trial of bevacizumab with chemotherapy in patients with advanced endometrial carcinoma did not show a statistically significantly increased PFS relative to historical controls when patients were analyzed as an entire cohort [13]. 

Preclinical studies have clearly demonstrated the activity of single-agent bevacizumab on a variety of tumor models, including endometrial carcinoma. In an orthotopic mouse model of endometrial cancer, bevacizumab significantly reduces tumor volume compared to control [14]. A previous study by our group also showed that bevacizumab inhibits the growth of endometrial cancer in a mouse xenograft model [15]. The anti-tumor activity of cediranib has been demonstrated in tumor xenograft models of various types of cancers including colon, lung, prostate, breast, and ovarian cancer [6,7,16]. In addition, cediranib decreases tumor vessel density and promotes vascular regression [6].

The vast majority of studies of antiangiogenic agents in endometrial cancer have focused on the inhibitory effects on the tumor vasculature. We hypothesized that therapeutic efficacy could also be derived from effects on the tumor cells. To date, cediranib has not been studied in combination with chemotherapy in endometrial cancer despite the documentation from GOG0229J that it has single-agent activity. The studies reported herein were designed to compare the impact of cediranib to bevacizumab to assess whether cediranib may also be effective as an anti-angiogenic and as an adjuvant to chemotherapy. Hence, we compared the efficacy of the two most active anti-angiogenic agents in endometrial cancer, bevacizumab and cediranib, on tumor cell survival and signaling. Whereas previous studies of bevacizumab activity were performed in vivo using xenograft models of endometrial cancer [14,15], herein we used immortalized endometrial cancer cell lines and patient-derived organoid models of endometrial cancer to specifically assess the impact of the agents on the tumor cells. Strengths and limitations of the various models of endometrial cancer have been recently reviewed [17]. Several well-characterized endometrial cancer cell lines are available for in vitro studies, including the poorly differentiated Hec50 and KLE cells. These cell lines are models of more aggressive endometrial cancer and harbor distinct mutations in *TP53* that are frequently detected in patient tumors [18]. This study also includes patient-derived organoid models of endometrial cancer whereby freshly resected tumor specimens are cultured in a three-dimensional model system [19,20]. As compared to patient-derived xenograft models, which also use fresh patient tumor tissue, patient-derived organoids are much more cost-effective with a higher rate of successful culture [17]. Both patient-derived organoid and xenograft models retain intratumoral heterogeneity, which is lacking in immortalized 2D cell lines [17]. A limitation of organoids as compared to xenografts is the absence of tumor vasculature. However, this provided a unique opportunity herein to study the tumor response to anti-angiogenic agents independent of vascular effects.

## 2. Results

### 2.1. Tyrosine Kinase Receptor Signaling Pathways Are Downregulated by Cediranib But Not Bevacizumab

**Figure 1:**
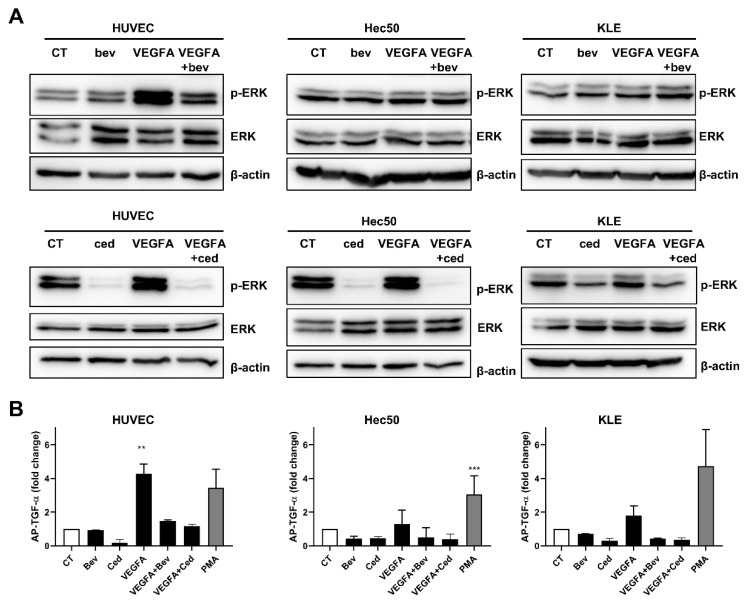
Impact of bevacizumab and cediranib on VEGFR signaling endothelial cells and endometrial cancer cells. (**A**) HUVEC, Hec50, and KLE cells were treated with vehicle control (CT), 1 µM bevacizumab (Bev), 100 ng/mL VEGF-A, or 1 µM cediranib (Ced) for 1 hr, followed by assessment of ERK1/2 phosphorylation at Thr202/Tyr204 or total ERK expression by Western blotting. β-actin: loading control. (**B**) Cells were transfected with the alkaline phosphatase (AP)-tagged ADAM17 substrate TGF-α, and treated as in (**A**) for 1 hr. The change in soluble AP-TGF-α was assessed and presented as fold change compared to control (CT). PMA (25 ng/mL) served as a positive control for induction of AP-TGF-α shedding. ** *p* < 0.01; *** *p* < 0.001 versus CT by one-way ANOVA with Tukey’s multiple comparisons test.

We first established the inhibitory potential of bevacizumab and cediranib on ligand-stimulated extracellular signal-related kinase (ERK) activation in human vascular umbilical endothelial cells (HUVEC) by Western blot (Figure 1A). In contrast to robust inhibition of ERK phosphorylation at Thr202/Tyr204 by both bevacizumab and cediranib in HUVEC cells, the phosphorylation form of ERK displayed no significant induction with VEGF-A treatment and no obvious change with the addition of bevacizumab in Hec50 and KLE cells. These data suggest that the intracellular signaling impact of VEGF-A is not as robust in these tumor cells as compared to the known impact of VEGF-A on the vasculature of the surrounding tumor microenvironment (Figure 1A, middle and right panels) [6].

Bevacizumab acts by selectively binding circulating VEGF, thereby inhibiting VEGFR1/2 activation [21]. Cediranib also inhibits VEGFR1/2/3 as well as PDGFRs and FGFR1 [6]. Previous studies have shown that activation of VEGFR2 by VEGF triggers a disintegrin and metalloprotease (ADAM) 17-dependent release of EGFR ligands such as transforming growth factor (TGF)-α in a variety of different cells [22]. To monitor the effect of bevacizumab and cediranib on activation of ADAM17, we stimulated cells expressing alkaline phosphatase (AP)-tagged TGF-α with VEGF-A. Phorbol 12-myristate 13-acetate (PMA), a well-known activator of ADAM17-mediated shedding events served as a positive control [23,24]. As anticipated, VEGF-A promoted a substantial increase in AP-TGF-α shedding in HUVEC cells, indicative of VEGFR2 activation (Figure 1B). This effect was blunted by both bevacizumab and cediranib. By contrast, VEGF-A did not significantly increase the shedding of AP-TGF-α in Hec50 and KLE cells (Figure 1B). These data are in alignment with the lack of ERK phosphorylation in Hec50 and KLE cells in response to VEGF-A. We confirmed that Hec50 and KLE cells express endogenous VEGF-A as well as VEGFRs, FGFRs, and PDGFRs (Supplemental Figure S1).

### 2.2. Cediranib But Not Bevacizumab Synergizes with Chemotherapy

**Figure 2:**
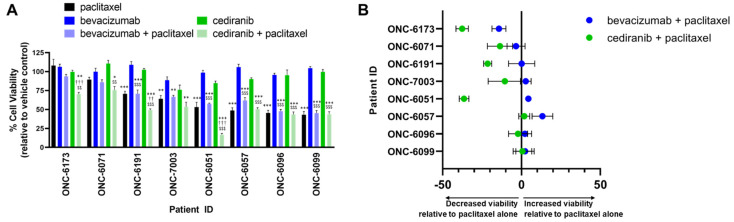
Effect of cediranib and bevacizumab on sensitivity to paclitaxel in patient-derived organoid cultures of primary endometrial tumors. Organoid cultures were treated with 10 nM paclitaxel, 1 µM bevacizumab, 1 µM cediranib, or the combination of paclitaxel with bevacizumab or cediranib for 72 hrs, followed by assessment of cell viability. (**A**) Data were calculated as the percent (%) cell viability as compared to vehicle control and plotted left-to-right by increasing sensitivity to single-agent paclitaxel. (**B**) The change in viability with the combination of paclitaxel with either bevacizumab or cediranib was calculated relative to paclitaxel alone. * *p* < 0.05, ** *p* < 0.01, *** *p* < 0.001 versus control; †† *p* < 0.01, ††† *p* < 0.001 versus paclitaxel alone; $$ *p* < 0.01, $$$ *p* < 0.001 versus anti-angiogenic agent alone (bevacizumab for bevacizumab + paclitaxel or cediranib for cediranib + paclitaxel treated samples) by ordinary one-way ANOVA with Tukey’s multiple comparisons test.

Based on the differential responses of bevacizumab and cediranib in our cell-based assays, we next examined the impact of these agents on cell viability in endometrial cancer models. Studies were first performed in eight patient-derived organoid models of endometrial cancer (Supplemental Table S1). The models represent the spectrum of endometrial cancer, from early-stage/grade endometrioid adenocarcinoma to stage IV serous adenocarcinoma. We previously reported that the combination of tyrosine kinase inhibitors (TKIs) or anti-angiogenic TKIs with the chemotherapy paclitaxel induces massive cell death via mitotic catastrophe [25,26,27]. We, therefore, screened the endometrial cancer organoid models for sensitivity to paclitaxel alone or in combination with bevacizumab or cediranib (Figure 2).

Compared to the untreated control, six of the eight patient-derived organoids exhibited a significant decrease in viability when treated with the paclitaxel, with a maximal decrease in viability of 57% by 72 h (Patient #ONC-6099) (Figure 2A). Both cediranib and bevacizumab as single agents had no impact on cell viability. The decrease in viability of combinatorial regimens was calculated relative to paclitaxel as a single agent (Figure 2B). In five of the models, cediranib but not bevacizumab further decreased cell viability when combined with paclitaxel as compared to treatment with paclitaxel alone (Figure 2B, ONC-6173, -6071, -6191, -7003, and -6051).

**Figure 3:**
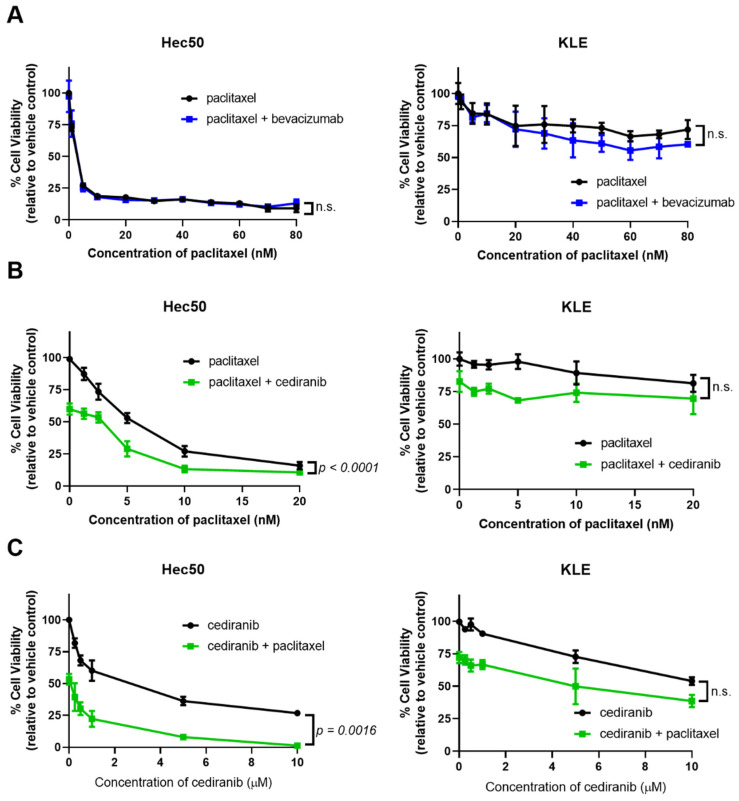
Cediranib but not bevacizumab increases sensitivity to paclitaxel in Hec50 endometrial cancer cells. (**A**) Hec50 (left) or KLE cells (right) were treated with increasing concentrations of paclitaxel in the absence or presence of 1 µM bevacizumab for 72 h; cell viability was determined using WST-1 assay relative to untreated control. (**B**) Hec50 (left) or KLE cells (right) were treated with increasing concentrations of paclitaxel in the absence or presence of 1 µM cediranib for 72 h; cell viability was determined using WST-1 assay. (**C**) Hec50 (left) or KLE cells (right) were treated with increasing concentrations of cediranib in the absence or presence of 5 nM paclitaxel for 72 h; cell viability was determined using WST-1 assay. For the combinatorial treatments in (**B**,**C**) (green lines), the points on the y-axis at 0 reflect treatment with either cediranib alone (**B**) or paclitaxel alone (**C**). For example, single-agent cediranib at 1 µM ((**B**), Hec50) results in a 40.0% decrease in cell viability as compared to untreated control. Statistical significance was assessed by two-way ANOVA with Sidak’s post-hoc test. n.s.: not significant.

Studies were then extended to well-characterized cell models of advanced endometrial cancer with varying baseline sensitivity to paclitaxel [26,27] (Figure 3). As in the organoid models, bevacizumab did not provide any additional cell killing when combined with paclitaxel (Figure 3A). Dose-response curves using either paclitaxel or cediranib in the presence of a set concentration of the opposing drug indicate synergy when the two agents are combined as compared to a single drug alone (Figure 3B,C). This trend was most pronounced in Hec50 cells, with no synergy observed in KLE cells treated with equivalent doses of the drugs (Figure 3B,C).

### 2.3. The Combination of Cediranib But Not Bevacizumab with Paclitaxel Increases the Percentage of Cells in Mitosis

**Figure 4:**
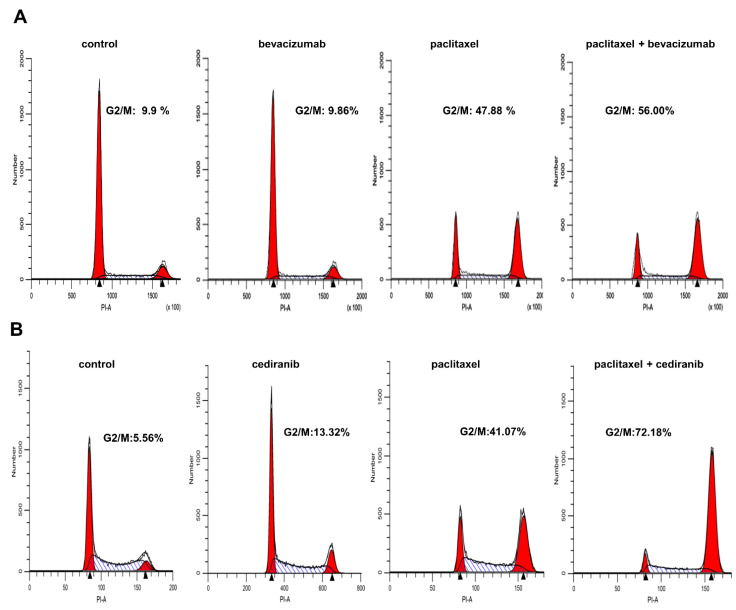
Comparison of the effect of cediranib and bevacizumab on cell cycle distribution in Hec50 cells treated with paclitaxel. (**A**) Cell cycle distribution in Hec50 cells treated with DMSO (Control), 14 nM paclitaxel, 1 μM bevacizumab, or a combination of paclitaxel and 1 μM bevacizumab for 24 h. (**B**) Cell cycle distribution for Hec50 cells treated with vehicle (DMSO), 14 nM paclitaxel, 1 μM cediranib, or a combination of 14 nM paclitaxel and 1 μM cediranib. Insets denote the percentage of cells in the G2/M phase of the cell cycle.

Flow cytometric measurements were performed to determine the effects of paclitaxel, cediranib, bevacizumab, and the combination treatment on cell cycle in Hec50 cells, which were more sensitive to the combination of cediranib and paclitaxel in Figure 3. Treatment with cediranib or bevacizumab alone had little effect on cell cycle distribution as compared to control treatment (Figure 4).

Paclitaxel is a microtubule stabilizing agent and thus is most effective in mitosis (M phase of the cell cycle). As expected, treatment with paclitaxel alone resulted in a marked shift in cells to the G2/M phase. The combination of paclitaxel and cediranib produced a profound increase in the accumulation of cells in mitosis as assessed by the percentage of cells in G2/M by flow cytometry compared to paclitaxel alone (Figure 4B). Yet there was minimal change in the percentage of cells in G2/M in bevacizumab + paclitaxel-treated cells (Figure 4A). These findings indicate that paclitaxel treatment in the presence of cediranib results in a significant enhancement of the G2/M population by flow cytometry—the predominance of the cells being in M.

To better understand the mechanism of the mitotic cell death induction by paclitaxel and cediranib, we next examined the expression and post-translational modification of critical regulators of the G2/M checkpoint including cyclin-dependent kinase 1 (cdc2) and cell division cycle 25C (CDC25C). The effect of gefitinib, a tyrosine kinase inhibitor specific for EGFR, in combination with paclitaxel serves as a positive control based upon our previous work that gefitinib and paclitaxel promote premature entry to mitosis, especially in Hec50 cells that are devoid of functional p53 [27].

**Figure 5:**
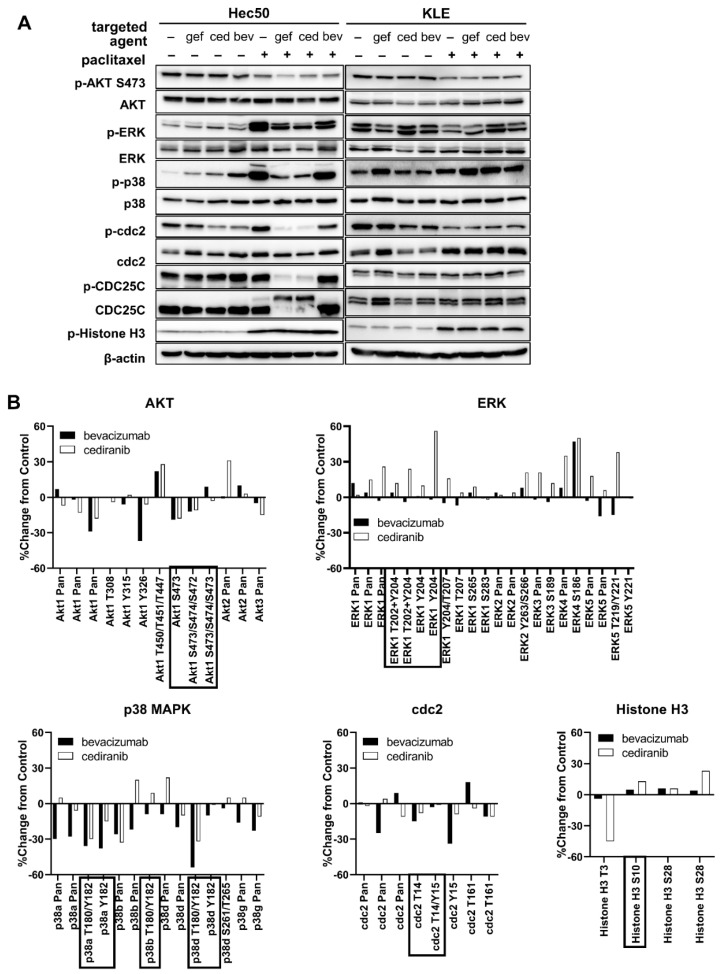
Molecular effects of anti-angiogenic agents on G2/M cell cycle controllers in endometrial cancer cell models. (**A**) Hec50 (left) or KLE cells (right) were treated with the indicated agents either alone or in combination with paclitaxel for 24 hrs, followed by assessment of cell cycle controllers by Western blotting. Drug concentrations: 10 μM gefitinib, 1 μM cediranib, 1 μM bevacizumab,14 nM paclitaxel. (**B**) Expression or phosphorylation of indicated proteins was assessed by Kinex™ KAM-1325 Phosphproteomic Antibody Microarray in Hec50 cells treated with 1 µM bevacizumab or 1 µM cediranib for 24 h. Data were calculated as the percent change from control (%CFC). Phosphosites corresponding to those queried in (**A**) are indicated with boxes. Pan: antibody total protein expression.

Treatment of Hec50 cells with cediranib in combination with paclitaxel decreased phosphorylation cdc2. De-phosphorylation of cdc2 is the final signaling step that opens the G2/M checkpoint, thereby allowing cells to enter mitosis. Cells that stop at G2/M have the potential to repair DNA prior to mitosis; however, when the checkpoint is abrogated as reflected by the dephosphorylation of cdc2, cells with damaged DNA enter mitosis and are more vulnerable to paclitaxel (Figure 5A).

The active form of CDC25C, a protein phosphatase responsible for dephosphorylating cdc2 (abrogating the G2/M checkpoint), was significantly increased by this treatment regimen as demonstrated by a loss of phosphorylation at Ser216 and a slower migrating band in the total CDC25C blot. These same signaling events were not observed with bevacizumab was combined with paclitaxel (Figure 5A), indicating a lack of G2/M checkpoint abrogation.

We also examined the effect of combinatorial treatment on KLE endometrial cancer cells, which we previously found to be resistant to paclitaxel + gefitinib [27]. Treatment with anti-angiogenic agents or gefitinib in combination with paclitaxel failed to override the G2/M cell cycle checkpoint, consistent with our previous findings. 

**Figure 6:**
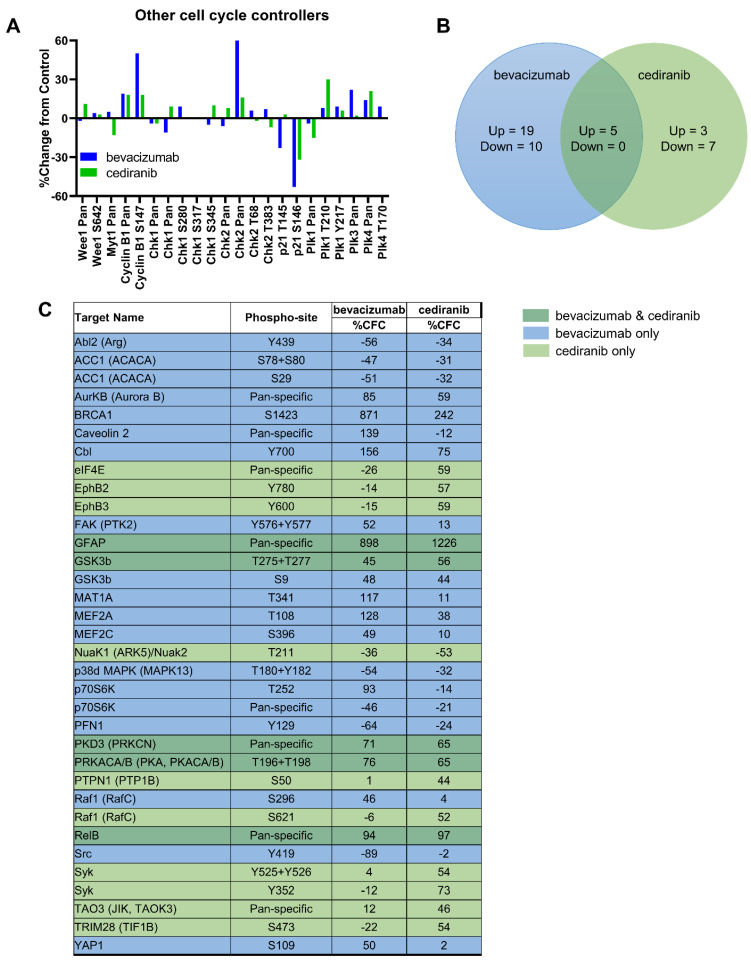
Bevacizumab regulates more signaling events than cediranib in endometrial cancer cells. Signaling events in response to single-agent bevacizumab or cediranib were analyzed by Kinex™ KAM-1325 Phosphproteomic Antibody Microarray after treatment of Hec50 cells with 1 µM bevacizumab or 1 µM cediranib for 24 h. (**A**) Depiction of changes in select cell cycle controllers. Data were calculated as the percent change from control (%CFC); “pan” indicates total protein expression. (**B**) Venn diagram of lead candidates in response to either bevacizumab or cediranib. Overlap indicates signaling events that were shared between the two treatment groups. Up = increased expression/phosphorylation; down = decreased expression/phosphorylation. (**C**) Table of all lead candidates identified based on the %CFC [28]. Negative %CFC indicates a decrease as compared to control. Full results are provided as Supplemental Table S2.

To better understand why bevacizumab has no impact on cell survival or cell cycle progression in cancer cells, we performed a phosphoproteomic array using a panel of over 800 phosphorylation sites [28]. We detected similar trends in Akt and ERK/MAPK signaling and G2/M cell cycle checkpoints in response to bevacizumab or cediranib as single agents in Hec50 cells (Figure 5B). Changes in other cell cycle controllers included in the phosphoproteomic array are depicted in Figure 6A. Interestingly, bevacizumab as a single agent promoted more signaling events than cediranib, with only five changes shared between the two treatment groups (Figure 6B,C). Additionally, cediranib largely promoted decreased phosphorylation of signaling molecules, the expected therapeutic effect of a multi-TKI, whereas bevacizumab both increased and decreased phosphorylation events. We hypothesize that increased phosphorylation on pro-growth signaling molecules in response to bevacizumab treatment indicates a potential signal of developing cellular resistance. 

## 3. Discussion

Angiogenesis plays a crucial role in tumor development and metastasis, and cancer cells frequently upregulate VEGF-A expression to promote angiogenesis. VEGF-A binds to VEGFR1 and VEGFR2, and it is the main stimulator of tumor growth and dissemination. Accordingly, inhibitors of angiogenesis have been tested in clinical trials for a range of cancers, with FDA approval for 11 agents that inhibit either VEGF ligands or receptors [29]. Bevacizumab, which has been FDA-approved for cervical and ovarian cancers, specifically binds to the VEGF-A protein, and thereby inhibits vessel growth in the tumor. Cediranib is a tyrosine kinase inhibitor that targets VEGFR1, 2, 3, PDGFRs, and FGFRs. Cediranib is thought to be effective in the prevention of tumor progression, not only by inhibiting VEGFR2 activity and angiogenesis but also by concomitantly inhibiting VEGFR3 activity and lymphangiogenesis.

While the role of anti-angiogenic agents on tumor vascularization and endothelial cell growth is well-documented [6,7,14,16], few studies have interrogated the impact of anti-angiogenic agents on the properties of tumor cells themselves. In the present study, our objective was to compare the impact of angiogenic inhibitors on endometrial cancer cells. First, we found that cancer cells were not significantly responsive to treatment with exogenous VEGF-A despite the expression of VEGFRs 1 and 3. Bevacizumab, a monoclonal antibody specifically raised against VEGF-A, had little impact on cell viability or cell cycle progression alone as shown in Figure 2, Figure 3 and Figure 4. In addition, bevacizumab did not synergize with chemotherapy, as we had previously found for other agents that target TKI signaling [25,26,27]. We provide evidence that synergy was related to the impact on the cell cycle when comparing bevacizumab, with its narrow range of activity, to a multi-targeted anti-angiogenic TKI such as cediranib. Thus, we propose that the ability of cediranib, but not bevacizumab, to inhibit multiple kinases and to dephosphorylate the G2/M gatekeeper cdc2 results in G2/M checkpoint abrogation, premature entry of tumor cells with damaged DNA into mitosis, and cell death through mitotic catastrophe. The significant enhancement of the percent of cells in G2/M when cells are treated with cediranib, and paclitaxel (as shown in Figure 4) is a potential mechanism of synergy. Another explanation for the enhanced response to cediranib versus bevacizumab is the robust expression of cediranib targets VEGFRs, PDGFRs, and FGFRs.

The phosphoproteomic array results reported are valuable as a readout of the numerous signaling events in response to bevacizumab and cediranib treatment at a single time point, 24 h (Figure 5 and Figure 6). The inhibition of phosphorylation of multiple targets related to cediranib activity likely underlies its therapeutic effects as a tyrosine kinase inhibitor [7]. In contrast, the phosphorylation events were more often induced after bevacizumab treatment (Figure 5). Though enhanced phosphorylation is most commonly associated with signaling activation and proliferative signals, in our study, it may suggest that cells treated with bevacizumab instead induce resistance pathways by 24 h. These specific phosphorylation events are worthy of future investigation as they shed light on therapeutic agents that could block the resistance phosphorylation events when added to bevacizumab. 

While no direct comparison of efficacy between cediranib and bevacizumab has been reported, we predicted cediranib would have greater benefit due to the additional blockade of all VEGFR isoforms as well as PDGF and FGF receptors. Isoforms of PDGFR and FGFR are expressed in endometrial tumors [30,31], with FGFR2 mutations occurring in ~13% of endometrial cancer cases [32]. We found that cediranib, as a multi-TKI inhibitor, had greater therapeutic effects, including blunting baseline ERK activation, synergizing with paclitaxel in organoid models and cell lines and the anticipated abrogation of the G2/M cell cycle checkpoint. 

A limitation of this study, and studies of immortalized cell lines and patient-derived organoid models in general, is the absence of tumor microenvironment features, including immune cells and vasculature. Conversely, a strength of using these models is the ability to determine the impact of bevacizumab and cediranib specifically on the tumor cells without the confounding influence of the tumor microenvironment. We attempted to perform all studies on organoids within two weeks of development (i.e., obtaining surgical specimens) in order to avoid the effects of tumor evolution in culture. Considering that the size of endometrial tumor specimens is typically very small as compared to other gynecologic cancer types (e.g., ovarian tumors), this approach precluded extensive molecular analyses. Therefore, as a companion, we assessed cell signaling using endometrial cancer cell lines. We did not extend studies to xenograft models of endometrial cancer since both bevacizumab and cediranib have been studied in this system previously by our group and others [6,7,14,15,16]. 

Findings from this preliminary study in preclinical models should be expanded to determine the mechanisms of differential sensitivity at the molecular level to cediranib, as evidenced by the lack of synergy when cediranib was combined with paclitaxel in KLE endometrial cancer cells (Figure 3) as well as some organoid models (Figure 2). We originally hypothesized that the mutations in p53, a well-established controller of the G1/S and G2/M cell cycle checkpoints, would predict for enhanced synergy. This hypothesis follows our recently published translational study of GOG-86P in which patients with mutations in *TP53* had significantly improved outcomes when bevacizumab was combined with chemotherapy as compared to other experimental agents [3]. However, the majority of endometrial organoid models used in this study had wild-type (WT) p53 (Figure 2), precluding a definitive analysis of the role of p53 in sensitivity.

## 4. Materials and Methods

### 4.1. Chemicals

Gefinitib, cediranib, bevacizumab, and paclitaxel were purchased from Selleck Chemicals, LLC (Houston, TX, USA) and suspended in DMSO.

### 4.2. Patient-Derived Organoid Models of Endometrial Cancer

All studies using human tissues have been approved by the University of Iowa Institutional Review Board (IRB), protocol #201809807. Patient tumor specimens were obtained within 30 min of surgical resection. Organoids were created per our protocol for ascites fluid [19], with modifications to digest tissue and isolate single cells. Specifically, freshly resected tissue was washed with 10 mL PBS and cut into small pieces. The minced fragments were collected in a 50 mL tube and digested in 5 mL AdDF+++ media (Advanced DMEM-F12 media with 1× Glutamax, 10 mM HEPES and Pen strep) supplemented with 2 U/mL Dispase II, 1 mg/mL collagenase P and 50 µg/mL Dnase I, then incubated at 37 °C for 0.5–1 h. Dissociated cells were filtered through a 40 μm cell strainer, centrifuged at 300× *g* for 10 min, washed twice with PBS, and pelleted. Erythrocytes were removed by incubating the dissociated cells with 2 mL red blood cell lysis buffer for 5 min at room temperature followed by an additional wash with 10 mL AdDF+++ and centrifugation at 300× *g* for 5 min. Finally, the cells were counted and embedded in Matrigel on ice and seeded on pre-warmed 24-well cell culture plates; 500 µL AdDF+++ media was added on the top of the Matrigel to each well.

### 4.3. Cell Culture

Hec50 cells were kindly provided by Dr. Erlio Gurpide (New York, NY, USA) [18] and grown in high-glucose Dulbecco’s Modified Eagle Medium (DMEM; Gibco Corporation, Gaithersburg, MD, USA) supplemented with 10% fetal bovine serum. KLE cells were purchased from ATCC and grown in RPMI-1640 medium supplemented with 10% fetal bovine serum. Human umbilical vascular endothelial cells (HUVEC) were purchased from ATCC and grown in EGMTM -2 MV Microvascular Endothelial Cell Growth Medium (2 BulletKit, Lonza, Alpharetta, GA, USA). To ensure rigor and reproducibility, the identity of all cell lines was confirmed using the CODIS genotyping test (Cat. No. CL1003, Bio-Synthesis, Lewisville, TX, USA).

### 4.4. Western Blot

Cells were collected and lysed with NP-40 lysis buffer with protease inhibitors. Lysates were analyzed for protein expression/phosphorylation as described previously [25,27]. The following antibodies were used at the indicated dilutions; all antibodies were purchased from Cell Signaling (Danvers, MA, USA): anti-p-AKT-S473 (1:1000, #4060), anti-AKT (1:1000, #4685), anti-p-p44/42 MAPK (Erk1/2) -Thr202/Tyr204 (1:1000, #4370), anti- p44/42 MAPK (Erk1/2) (1:1000, #4695), anti-p-p38 MAPK-Thr180/Tyr182 (1:1000, #9211), anti-p38 MAPK (1:1000, #8690), anti-p-cdc2-Tyr15 (1:1000, #4539), anti-cdc2 (1:1000, #9116), anti-p-CDC25C-Ser216 (1:1000, #4901), anti-CDC25C(1:1000, #4688), anti-p-Histone H3-ser10 (1:1000, #53348).

### 4.5. Cell Viability Assay

#### 4.5.1. Organoids

Viability of the tumor organoids following drug treatment was performed as previously described [19]. Briefly, patient-derived organoids were collected with organoid harvesting solution (Cultrex, Trevigen, Gaithersburg, USA), and then digested to single cells with TrypLE Express supplemented with 4 µL 10 mg/mL DNAse I stock and 4 µL 10 mM Y-27632 stock. Single cells were suspended in AdDF+++ medium with 10% Matrigel and seeded at a density of 10,000 cells/well (50 μL/well) in an ultra-low attachment 96-well U-bottom white plate. After 24 h, cells were exposed to paclitaxel (10 nM), bevacizumab (1 µM), or cediranib (1 µM) for 72 h at 37 °C. At the end of incubation, an equal volume of CellTiter-Glo 3D reagent (Promega) was added to each well and incubated for 25 min at room temperature. The luminescence was measured using the Gen5 Microplate Reader (BioTek, Winooski, VT, USA). All the tests were conducted in triplicate and data normalized to untreated control (set at 100% viability).

#### 4.5.2. Cell Lines

Cell viability was determined by WST-1 assay as previously described [26]. Briefly, Hec50 and KLE cells were seeded into 96-well plates (10,000 cells per well) for 24 h and then cultured with increased concentrations of the drugs for an additional 72 h. Cell viability was evaluated using the cell proliferation reagent WST-1 (Roche, Pleasanton, Germany) according to the manufacturer’s protocol. The absorbance was measured with a micro-plate reader (Bio-Rad Laboratories, Inc., Hercules, CA, USA). All the tests were conducted in triplicate and data were normalized to untreated control (set at 100% viability).

### 4.6. Cell Cycle Analysis

Cell cycle analysis was performed by flow cytometry as described previously [25]. An equal number of cells were plated in 10 cm plates and treated with different drugs for 24 h as for cell viability assays. Cell pellets were harvested and suspended separately in Krishan’s solution (3.8 mM sodium citrate, 0.014 mM propidium iodide, 1% NP-40, and 2.0 mg/mL RNase A). Cell suspensions were analyzed using a FacScan Flow Cytometer (Becton, Dickinson and Company, San Jose, CA, USA) and data were analyzed by CellQuest software version 3.3.

### 4.7. Shedding Assay

HUVEC, Hec50, and KLE cells were grown to 70–80% confluence and serum-starved in Opti-MEM for one hour prior to transfection. Transfection of 1 µg alkaline phosphatase (AP)-tagged transforming growth factor (TGF)-α were performed according to manufacturer’s protocols using Lipofectamine2000 (Invitrogen, Carlsbad, CA, USA). Cells were starved for at least four hours before the stimulation. Recombinant human vascular endothelial growth factor (VEGF-A) was obtained from R&D Systems (Minneapolis, MN, USA) and used at a concentration of 100 ng/mL. The concentration of bevacizumab was 1 µg/mL. PMA (phorbol-12-myristate-13-acetate) and the AP-tagged TGF-α plasmid have been previously published [33]. Evaluation of AP activity was determined by the colorimetric assay as described previously [34]. Briefly, the ratio between the total AP activity in the supernatant and the total AP activity in the cell lysate plus the supernatant was computed for normalization. The presented ratios reflect the relative proteolytic activity of a given sheddase toward the AP-tagged ligand TGF-α. No AP activity was detected in the conditioned media of untransfected cells.

### 4.8. Phosphoproteomic Microarray

Hec50 cells were plated in 15 cm plates and treated with DMSO, 1µM bevacizumab, or 1µM cediranib for 24 h. Cell pellets were collected and lysed with Kinexus Lysis Buffer with chemical cleavage. The microarray assay and data analysis were performed with Kinex™ KAM-1325 Antibody Microarray Kit by Kinexus Bioinformatics Corporation (Vancouver, BC, Canada). To qualify as a lead, the percent change from control (%CFC) value should be at least 45% higher or lower with fluorescent signals that were at least 1000 counts [28].

### 4.9. Reverse Transcription and Quantitative Polymerase Chain Reaction

Total RNA was purified from Hec50co and KLE cells using the RNeasy Plus RNA purification kit (QIAGEN, Germantown, MD, USA). Yield and purity were assessed on a Trinean DropSense 16 spectrophotometer and an Agilent Model 2100 Bioanalyzer in the Genomics Division of the Iowa Institute of Human Genetics (IIHG). The mean RNA Integrity Number (RIN) was 9.5 [35]. Next, 500 ng of QC qualified total RNA from each cell line was reverse transcribed using oligo-dT primers in the SuperScript III kit following the manufacturer’s recommendations (Invitrogen). The resulting cDNAs were then amplified in the presence of SYBR Green (Thermo Fisher, Waltham, MA, USA) on an Applied Biosystems Model 7900HT platform in the Genomics Division of the Iowa Institute of Human Genetics (IIHG). Locus-specific primers are shown below (Table 1). Raw expression values (Ct) were normalized (ΔCt) [36] against the endogenous 18S rRNA control.

### 4.10. Statistical Analysis

Data were analyzed using GraphPad Prism software (GraphPad Software Version 0.0.0 (121), San Diego, CA, USA). Statistical significance of differences was determined using one-way ANOVA with Sidak’s post-hoc test (for comparison of significance between two treatment groups with multiple independent treatment concentrations; Figure 3) or two-way ANOVA with Tukey’s post-hoc test (for comparisons of means of multiple groups; Figure 2A). The normal distribution of data was assessed using the likelihood test. All values are expressed as mean ± standard deviation (SD) of at least three independent experiments unless otherwise indicated.

## 5. Conclusions

In conclusion, the standard of care for endometrial cancer has not changed beyond chemotherapy, the incidence is on the rise due to the obesity epidemic, and outcomes are worse now than in the 1970s. The data presented in this report are designed to identify agents that should be advanced into clinical trials to improve outcomes for women with endometrial cancer. Based on our findings, we put forth that cediranib should be investigated in future clinical trials in endometrial cancer in the upfront setting in combination with chemotherapy. Specifically, our data suggest that the anti-angiogenic agent cediranib may have additional advantages over bevacizumab as an anti-cancer therapeutic due to its dual effects on tumor cells and vasculature. Our use of endometrial tumor tissue cultured as functional three-dimensional organoids provides highly translational information on the efficacy of anti-angiogenic agents in tumor cells. These data set the stage for future clinical trials to evaluate cediranib in combination with chemotherapy as a treatment for women with endometrial cancer.

## Figures and Tables

**Table 1:** Primer sequences.

Locus	Primer Sequences	Amplicon	Tm (°C) *
VEGFA	For: GGGCAGAATCATCACGAAGT	269 bp	55.0
	Rev: AGGAAGCTCATCTCTCCTATGT		54.7
VEGFR1	For: GGACAGTAGAAAGGGCTTCATC	251 bp	55.2
	Rev: CAGGGTAACTCCAGGTCATTTG		55.4
VEGFR2	For: GTGGTCTCTCTGGTTGTGTATG	235 bp	55.0
	Rev: CCTCCACACTTCTCCATTCTTC		55.1
VEGFR3	For: CGAAAGTGCATCCACAGAGA	239 bp	54.9
	Rev: AGAGAGAAGATCTCCCAGAGAAG		54.9
FGFR1	For: AGGAACTTTTCAAGCTGC		
	Rev: CATCATGTACAGCTCGTTG		
FGFR2	For: ATGAGGAATACTTGGACCTC		
	Rev: TTAACACTGCCGTTTATGTG		
FGFR3	For: GAAGATGCTGAAAGACGATG		
	Rev: GCAGGTTGATGATGTTTTTG		
FGFR4	For: CTGAGGACAATGTGATGAAG		
	Rev: CCGTTGCTGGTTTTCTTCTTATAG		
PDGFRA	For: GACTTTCGCCAAAGTGGAGGAG	121 bp	58.0
	Rev: AGCCACCGTGAGTTCAGAACGC		62.0
PDGFRB	For: TGCAGACATCGAGTCCTCCAAC	108 bp	59.0
	Rev: GCTTAGCACTGGAGACTCGTTG		57.8
18S rRNA	For: AACTTTCGATGGTAGTCGCCG	104 bp	57.3
	Rev: CCTTGGATGTGGTAGCCGTTT		57.6

* Tm calculated in the presence of 1.5 mM MgCl_2_.

## Data Availability

Data are contained within the article and Supplementary Material.

## Supplementary Materials

The following are available online at https://www.mdpi.com/article/10.3390/ph14070682/s1: Figure S1: Expression of bevacizumab and cediranib targets, Table S1: Patient characteristics for endometrial cancer organoids, Table S2: Raw data for phosphoproteomic array.
