# Supplementary material for: Advantages of Tyrosine Kinase Anti-Angiogenic Cediranib over Bevacizumab: Cell Cycle Abrogation and Synergy with Chemotherapy
Source: Pharmaceuticals (Basel). 2021 Jul 16;14(7):682. doi: 10.3390/ph14070682 (PMC8308742; doi:10.3390/ph14070682)
Supplement: Supplementary file 1 [file pharmaceuticals-14-00682-s001.zip › Supplemental Figure S1.pdf]

Supplemental Figure S1

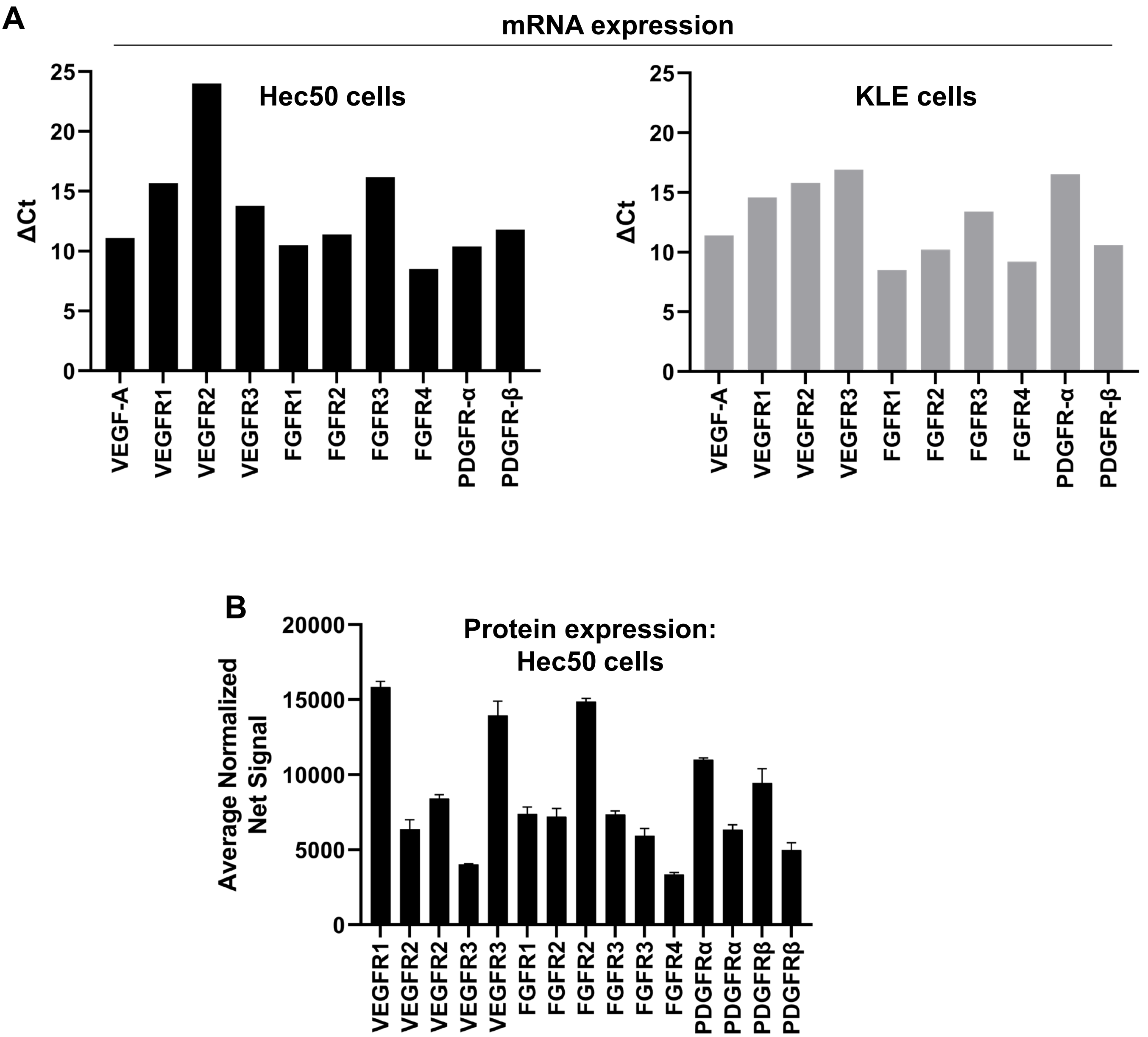

Supplemental Figure S1. Expression of bevacizumab and cediranib targets in endometrial cancer cells. (A) qRT-PCR for the bevacizumab ligand VEGF-A and cediranib targets VEGFR1, 2 and 3, FGFR1, 2 and 3, and PDGFR-*α* and PDGFR-β in Hec50 and KLE endometrial cancer cells. The lower the ΔCt value, the higher the expression level. A ΔCt value of 10-15 is indicative of robust mRNA expression, whereas values over 22 are very low expression (e.g., VEGFR2). N=3 (B) Protein levels in Hec50 cells as determined by Kinex™ KAM-1325 Phosphproteomic Antibody Microarray. The repeat entries for certain receptors (e.g., VEGFR2) are reflective of different antibodies against different epitopes. Data were normalized to internal controls and calculated as the average normalized net signal (N=2). See also **Supplemental Table S2**.
