# Supplementary material for: Advantages of Tyrosine Kinase Anti-Angiogenic Cediranib over Bevacizumab: Cell Cycle Abrogation and Synergy with Chemotherapy
Source: Pharmaceuticals (Basel). 2021 Jul 16;14(7):682. doi: 10.3390/ph14070682 (PMC8308742; doi:10.3390/ph14070682)
Supplement: Supplementary file 1 [file pharmaceuticals-14-00682-s001.zip › Supplemental Table S1.pdf]

Table S1: Patient Characteristics for endometrial cancer organoids.

IHC: immunohistochemistry; WT: wild-type. N.D.: Not determined

Patient ID	Diagnosis	Stage	Age (years)	p53 status	
				Sequence	IHC
ONC-6173	Endometrioid adenocarcinoma	IA	50	P278T	Overexpressed
ONC-6071	Endometrioid adenocarcinoma	IA	47	WT	Heterogenous expression
ONC-6191	Endometrioid adenocarcinoma	IB	67	WT	N.D.
ONC-7003	Mixed serous/endometrioid adenocarcinoma	IA	71	N.D.	N.D.
ONC-6051	Endometrioid adenocarcinoma	IA	69	WT	N.D
ONC-6057	Serous adenocarcinoma	IVB	70	WT	Overexpressed
ONC-6096	Endometrioid adenocarcinoma	IA	60	WT	Null
ONC-6099	Serous adenocarcinoma	IVB	57	Frameshift mutant	Null
